# Novel Cases of Non-Syndromic Hearing Impairment Caused by Pathogenic Variants in Genes Encoding Mitochondrial Aminoacyl-tRNA Synthetases

**DOI:** 10.3390/genes15070951

**Published:** 2024-07-19

**Authors:** María Domínguez-Ruiz, Margarita Olarte, Esther Onecha, Irene García-Vaquero, Nancy Gelvez, Greizy López, Manuela Villamar, Matías Morín, Miguel A. Moreno-Pelayo, Carmelo Morales-Angulo, Rubén Polo, Martha L. Tamayo, Ignacio del Castillo

**Affiliations:** 1 Servicio de Genética, Hospital Universitario Ramón y Cajal, IRYCIS, 28034 Madrid, Spain; 2 Centro de Investigación Biomédica en Red de Enfermedades Raras (CIBERER), 28034 Madrid, Spain; 3 Instituto de Genética Humana, Facultad de Medicina, Pontificia Universidad Javeriana, Bogotá 110231, Colombia; 4 Servicio de Genética, Hospital Universitario Marqués de Valdecilla, IDIVAL, 39008 Santander, Spain; 5 Programa de Doctorado en Biología, Escuela de Doctorado de la Universidad Autónoma de Madrid, 28049 Madrid, Spain; 6 Servicio de Otorrinolaringología, Hospital Universitario Marqués de Valdecilla, IDIVAL, 39008 Santander, Spain; 7 Facultad de Medicina, Universidad de Cantabria, 39005 Santander, Spain; 8 Servicio de Otorrinolaringología, Hospital Universitario Ramón y Cajal, 28034 Madrid, Spain

**Keywords:** aminoacyl-tRNA synthetases, mitochondria, *KARS1*, *HARS2*, *LARS2*, hearing loss, DFNB89, Perrault syndrome

## Abstract

Dysfunction of some mitochondrial aminoacyl-tRNA synthetases (encoded by the *KARS1*, *HARS2*, *LARS2* and *NARS2* genes) results in a great variety of phenotypes ranging from non-syndromic hearing impairment (NSHI) to very complex syndromes, with a predominance of neurological signs. The diversity of roles that are played by these moonlighting enzymes and the fact that most pathogenic variants are missense and affect different domains of these proteins in diverse compound heterozygous combinations make it difficult to establish genotype–phenotype correlations. We used a targeted gene-sequencing panel to investigate the presence of pathogenic variants in those four genes in cohorts of 175 Spanish and 18 Colombian familial cases with non-DFNB1 autosomal recessive NSHI. Disease-associated variants were found in five cases. Five mutations were novel as follows: c.766C>T in *KARS1*, c.475C>T, c.728A>C and c.1012G>A in *HARS2*, and c.795A>G in *LARS2*. We provide audiograms from patients at different ages to document the evolution of the hearing loss, which is mostly prelingual and progresses from moderate/severe to profound, the middle frequencies being more severely affected. No additional clinical sign was observed in any affected subject. Our results confirm the involvement of *KARS1* in DFNB89 NSHI, for which until now there was limited evidence.

## 1. Introduction

Aminoacyl-tRNA synthetases (ARS) are key enzymes in ensuring the accuracy of translation. They catalyze the attachment of amino acids to the acceptor arm of tRNAs in a specific manner, so that each enzyme attaches only one and the same amino acid to all tRNAs with an appropriate anticodon, according to the genetic code [1]. Some enzymes also have a proofreading activity, which targets and cleaves misacylated tRNAs. In addition to these housekeeping tasks, they play a diversity of noncanonical roles in regulating transcription, RNA splicing, translation and apoptosis, thus participating in a variety of physiological processes [2]. ARS enzymes also differ structurally. They are classified into two types according to the structure of their catalytic domains: the Rossman fold domain (Class I) or the seven β-strands domain (Class II). In addition, different non-catalytic domains allow ARS to play those noncanonical roles. Most Class I enzymes are monomeric, whereas Class II enzymes are dimeric (a majority) or multimeric [1,2]. 

The human genome contains 37 genes encoding ARS. These include genes that specifically code for the cytosolic enzymes (e.g., *LARS1*, for the cytosolic leucyl-tRNA synthetase) or for the mitochondrial enzymes (e.g., *LARS2*, for the mitochondrial leucyl-tRNA synthetase). In only two cases, both the cytosolic and mitochondrial enzymes are encoded by the same gene (*KARS1*, for lysyl-tRNA synthetase, and *GARS1*, for glycyl-tRNA synthetase). This is possible because alternative splicing of specific exons ensures that the polypeptide contains the appropriate sorting signals to be directed to the correct cell compartment [1,2].

Pathogenic variants in the diverse genes encoding ARS cause up to 56 different disorders (46 with an autosomal recessive (AR) inheritance, 10 with autosomal dominant (AD) inheritance). AD inheritance is typically associated with late-onset, progressive and neurodegenerative diseases. On the contrary, AR inheritance is usually associated with early-onset, severe and multi-system disorders [3].

Dysfunction of four mtARS results in hearing impairment (HI), either as the only clinical sign (non-syndromic hearing impairment, NSHI) or as part of complex syndromes. Biallelic pathogenic variants in *KARS1* can result in NSHI (DFNB89 type), or in HI associated with leukoencephalopathy and other clinical signs [4,5]. Biallelic pathogenic variants in *NARS2* (encoding mitochondrial ARS for asparagine) can produce NSHI (DFNB94 type), or complex syndromes including, among other signs, developmental delay, epilepsy and HI [6,7]. Biallelic pathogenic variants in either *HARS2* (mtARS for histidine) or *LARS2* (mtARS for leucine) result in Perrault syndrome, which associates HI with ovarian dysfunction in females, and manifests as NSHI in males [8,9]. Patients may develop a variety of neurological signs (polyneuropathy, ataxia, dysarthria, etc.) (Figure S1 and Table S1).

We have investigated the presence of pathogenic variants in those four genes in Spanish and Colombian cohorts of patients with autosomal recessive NSHI. We report five novel cases who carry nine different pathogenic variants, five of them novel, which expands the still small spectrum of variants that are involved in HI caused by mtARS dysfunctions. Their epidemiological contribution to autosomal recessive NSHI and their complex genotype–phenotype correlations are discussed.

## 2. Materials and Methods

### 2.1. Human Subjects

We enrolled a cohort of 316 Spanish unrelated familial cases of autosomal recessive NSHI. All cases in this cohort had at least two affected siblings with unaffected parents. Before this work, a preliminary screening of the *GJB2* gene (coding region and splice sites) by Sanger sequencing, as well as testing for the common del(*GJB6*-D13S1830) and del(*GJB6*-D13S1854) deletions in the DFNB1 locus, revealed disease-associated genetic variants in 141 families. The remaining 175 families were investigated in this study. After approval by the Ethical Committee of Hospital Universitario Ramón y Cajal (in accordance with the 1964 Declaration of Helsinki), written informed consent was obtained from all participating subjects.

We also included in this study a cohort of 18 Colombian unrelated familial cases of autosomal recessive NSHI, with at least two affected siblings and unaffected parents, and in whom DFNB1 mutations had been excluded. Their study was approved by the Ethical Committee of the School of Medicine of Pontificia Universidad Javeriana (in accordance with the 1964 Declaration of Helsinki), and written informed consent was obtained from all participating subjects.

Hearing was examined by pure-tone audiometry, testing for air conduction (frequencies 250–8000 Hz) and bone conduction (frequencies 250–4000 Hz). Hearing impairment was classified as mild (21–40 dB HL), moderate (41–70 dB HL), severe (71–95 dB HL) and profound (>95 dB HL), according to the pure-tone average (PTA) threshold levels at 0.5, 1, 2 and 4 kHz. Other clinical data were obtained from the patients’ medical histories, and through reexamination after a genetic diagnosis was established.

### 2.2. DNA Purification and Sequencing

DNA was extracted from peripheral blood samples by using the Chemagic Magnetic Separation Module I automated system (Chemagen, Baesweiler, Germany).

Targeted Massively Parallel DNA Sequencing was performed by using the OTO-NGS v2 gene panel, which was developed in our laboratory. It contains 117 genes that are known to be involved in NSHI (including *KARS1*, *HARS2*, *LARS2* and *NARS2*), and it is based on the IDT probe capture system [10]. Captured enriched libraries were sequenced on the Illumina NextSeq 550 platform (Illumina, Inc., San Diego, CA, USA). Sequence data were mapped against human genome GRCh37/hg19 reference sequence and analyzed using Sophia Genetics’ software v5.10.42.1 (Sophia Genetics, Rolle, Switzerland) to annotate and prioritize single nucleotide variants and copy number variants.

Sanger DNA sequencing was used to confirm those variants that were considered to be disease-associated and to study their segregation in the pedigrees. Primers and PCR conditions for the amplicons containing those variants are shown in Table 1. Sanger DNA sequencing was performed in an ABI Prism 3100 Avant Genetic Analyzer (Applied Biosystems, Waltham, MA, USA).

### 2.3. Assessment of Pathogenicity of DNA Variants

Pathogenicity of DNA variants was assessed according to the guidelines from the American College of Medical Genetics and Genomics and the Association for MolecularPathology (ACMG/AMP) [11], as implemented by Varsome [12], using GRCh38 as human reference genome. Scores were subsequently modified manually to delete criterion PP2 and to take into consideration criterion PM3, as recommended in the disease-specific ACMG/AMP guidelines for hearing loss [13].

## 3. Results

We investigated 193 familial cases of non-DFNB1 autosomal recessive NSHI (175 Spanish, 18 Colombian) for the presence of pathogenic variants in genes encoding ARS. Targeted Massively Parallel DNA Sequencing of the OTO-NGS v2 gene panel was performed on the propositus of every case. Disease-associated variants were found in *KARS1* (2 cases), *HARS2* (2 cases) and *LARS2* (1 case). Their segregation in each family was studied by Sanger sequencing, as follows.

### 3.1. KARS1

**Figure 1:**
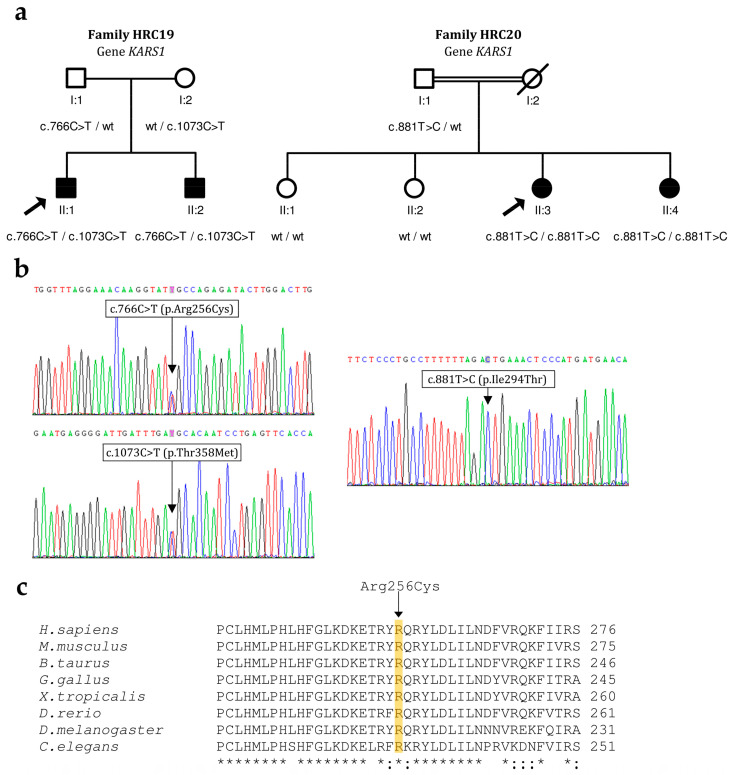
Pathogenic variants that were found in *KARS1* in this study. (**a**) Pedigrees showing the segregation of variants. Arrow indicates propositus. (**b**) Electropherograms from subject HRC19 II:1 (left panel) and from subject HRC20 II:3 (right panel). (**c**) Alignment of KARS1 protein orthologous sequences from human and seven other animal species. Asterisks indicate identical residues across all sequences; colons, conserved positions (residues of strongly similar properties); periods, semi-conserved positions (residues of weakly similar properties). Sequence accession numbers: *Homo sapiens* (NP_001123561.1); *Mus musculus* (NP_001124340.1); *Bos taurus* (XP_010812603.1); *Gallus gallus* (NP_001025754); *Xenopus tropicalis* (XP_012816251.2); *Danio rerio* (NP_001002386.1); *Drosophila melanogaster* (NP_572573.1); *Caenorhabditis elegans* (NP_495454.1).

**Figure 2:**
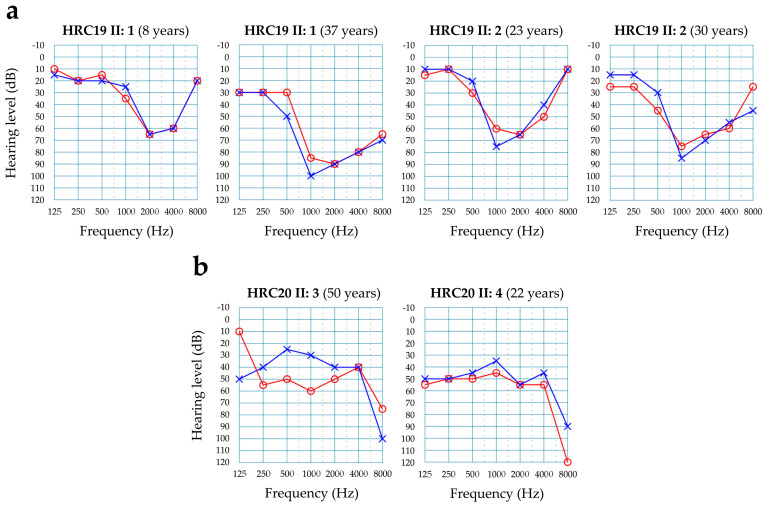
Audiograms from subjects with *KARS1* variants causing sensorineural hearing loss. Only results for air conduction are shown. Red line and circles, right ear. Blue line and crosses, left ear. (**a**) Affected subjects from family HRC19. Audiograms at different ages, to illustrate mild progression of the hearing loss. (**b**) Affected subjects from family HRC20.

In Spanish family HRC19, the two affected brothers were compound heterozygous for c.766C>T (p.Arg256Cys) in exon 7 of *KARS1* and c.1073C>T (p.Thr358Met) in exon 9, whereas their father carried c.766C>T and their mother carried c.1073C>T (Figure 1). The family had no siblings with normal hearing. The c.1073C>T variant has been reported previously in the homozygous state in the only affected subject of an Iranian family, who showed neurological signs but not hearing loss at the age of testing [14]. The c.766C>T missense variant is novel. It affects an evolutionarily conserved residue in the helix of dimerization of the KARS1 polypeptide (Figure 1c) [15]. According to the ACMG criteria, the variant is classified as likely pathogenic (Table 2). Of note, a c.774A>T (p.Arg258Ser) pathogenic variant, which was reported in two unrelated cases of hearing loss and neurological features [14], affects the highly conserved arginine-258 residue, very close to the arginine-256 residue in the helix of dimerization. In both affected brothers of family HRC19, the hearing loss had a prelingual onset. All audiograms show a ‘cookie-bite’ shape (Figure 2a). As documented by audiograms at different ages, their hearing loss is progressing mildly (about 1 dB/year) from moderate to severe. No other clinical signs were found in their medical histories or in their reexamination at the time of genetic diagnosis (ages 37 and 30 years, respectively).

In Spanish family HRC20, parents were first cousins, the mother having died before the family was referred for this genetic study. The two affected sisters were homozygous for c.881T>C (p.Ile294Thr) in exon 8 of *KARS1*, whereas the father was a heterozygous carrier (Figure 1). Two other sisters, with normal hearing, did not carry the variant. The c.881T>C variant was reported in the compound heterozygous state with c.1760C>T (p.Thr587Met) in a 35-years old male with congenital hearing loss and neurological signs [16]. In family HRC20, the hearing loss was postlingual and moderate, the shape of the audiograms being similar in the two sisters (Figure 2b). The onset occurred at age 15 years in the youngest sibling (II:4), and much later in life, first noticed at age 40 years, in her older sister (II:3). Both suffered a sudden rapid progression to a profound HI that required bilateral cochlear implantation (II:4 at ages 29 years (right ear) and 34 years (left ear); II: 3 at age 54 years). At age 44 years, subject II:3 began to complain of fluctuating dysgeusia (metallic taste). No other clinical signs were found in their medical histories.

### 3.2. HARS2

**Figure 3:**
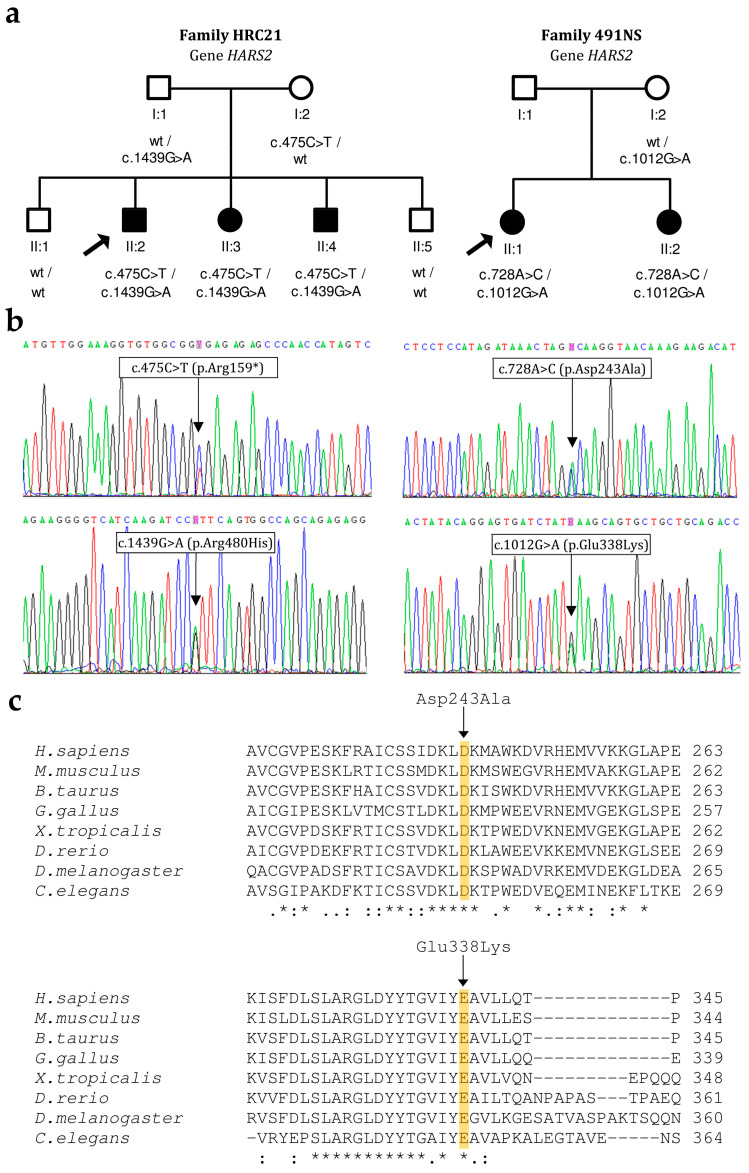
Pathogenic variants that were found in *HARS2* in this study. (**a**) Pedigrees showing the segregation of variants. Arrow indicates propositus. (**b**) Electropherograms from subject HRC21 II:2 (left panel) and from subject 491NS II:1 (right panel). (**c**) Alignment of HARS2 protein orthologous sequences from human and seven other animal species. Asterisks indicate identical residues across all sequences; colons, conserved positions (residues of strongly similar properties); periods, semi-conserved positions (residues of weakly similar properties). Sequence accession numbers: *H. sapiens* (NP_036340.1); *M. musculus* (NP_542367.1); *B. taurus* (XP_010805666.1); *G. gallus* (XP_040538754.1); *X. tropicalis* (XP_031754386.1); *D. rerio* (NP_001289185); *D. melanogaster* (NP_728180.1); *C. elegans* (NP_001023374.1).

**Figure 4:**
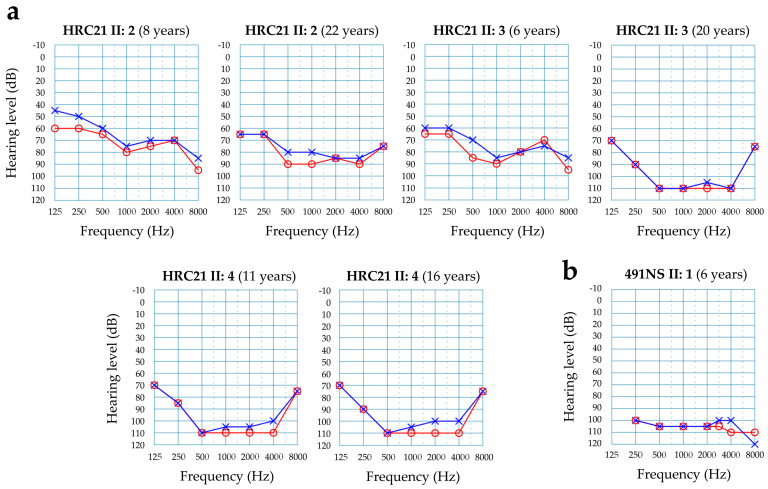
Audiograms from subjects with *HARS2* variants causing sensorineural hearing loss. Only results for air conduction are shown. Red line and circles, right ear. Blue line and crosses, left ear. (**a**) Affected subjects from family HRC21. Audiograms at different ages, to illustrate the evolution of the hearing loss. (**b**) Affected subject from family 491NS.

In Spanish family HRC21, the three affected siblings (two males, one female) were compound heterozygous for c.475C>T (p.Arg159*) in exon 5 of *HARS2* and c.1439G>A (p.Arg480His) in exon 12, whereas their father carried c.1439G>A and their mother carried c.475C>T (Figure 3). Two other siblings, with normal hearing, did not carry any variant. The c.475C>T nonsense variant is novel and pathogenic, as the exon is present in a biologically relevant transcript, it is predicted that the mutated transcript will undergo nonsense-mediated decay and there are previous reports that loss-of-function variants in this gene are pathogenic. The c.1439G>A missense variant was reported previously in the compound heterozygous state with other pathogenic variants in three unrelated cases with non-syndromic hearing loss [17]. In the three affected siblings of family HRC21, hearing loss had a prelingual onset. At the start, it was moderate-severe with a slightly downsloping audiogram, and later it evolved to become profound with a ‘cookie-bite’ audiogram (Figure 4a). No neurological signs were found in any of the three affected subjects. Currently, at age 21 years old, menstrual cycles are normal in the only affected female.

In Colombian family 491NS, the two affected sisters were compound heterozygous for c.728A>C (p.Asp243Ala) in exon 7 of *HARS2* and c.1012G>A (p.Glu338Lys) in exon 10, whereas their mother carried c.1012G>A (Figure 3). Their father could not be tested. The family had no siblings with normal hearing. Both missense variants are novel. They affect evolutionarily conserved residues in the catalytic domain of the HARS2 polypeptide (Figure 3c) [15]. According to the ACMG criteria, these variants are classified as likely pathogenic (c.728A>C) and pathogenic (c.1012G>A) (Table 2). Both sisters had prelingual profound hearing impairment (Figure 4b). Menarche occurred at normal ages (II:1, age 14 years; II:2, age 12 years). To date, no premature cessation of menstrual cycles has occurred, and no neurological signs have manifested (current ages: 24 and 22 years, respectively).

### 3.3. LARS2

**Figure 5:**
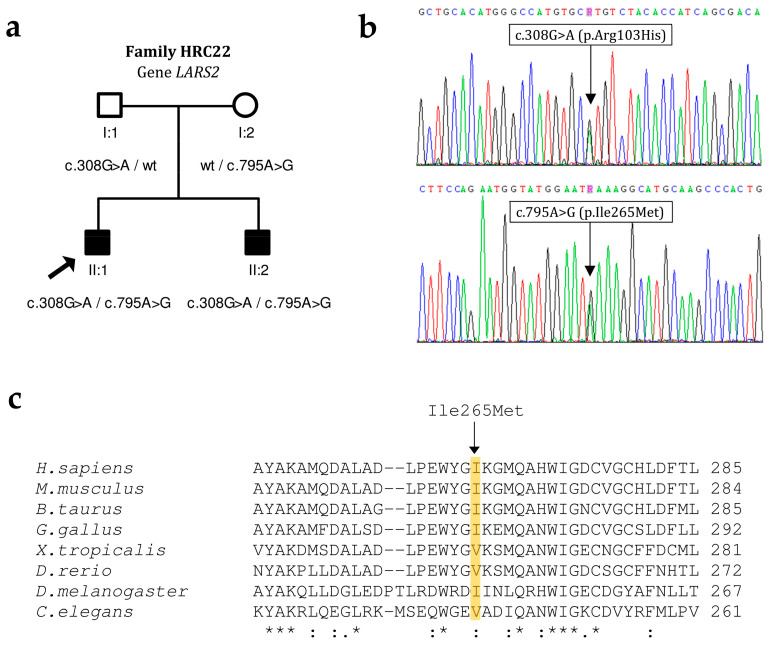
Pathogenic variants that were found in *LARS2* in this study. (**a**) Pedigree showing the segregation of variants. Arrow indicates propositus. (**b**) Electropherograms from subject HRC22 II:1. (**c**) Alignment of LARS2 protein orthologous sequences from human and seven other animal species. Asterisks indicate identical residues across all sequences; colons, conserved positions (residues of strongly similar properties); periods, semi-conserved positions (residues of weakly similar properties). Sequence accession numbers: *H. sapiens* (NP_056155.1); *M. musculus* (NP_001335096.1); *B. taurus* (XP_059735480.1); *G. gallus* (XP_040521344.1); *X. tropicalis* (XP_031759687.1); *D. rerio* (NP_001099171.1); *D. melanogaster* (NP_647932.1); *C. elegans* (NP_001021875.1).

**Figure 6:**
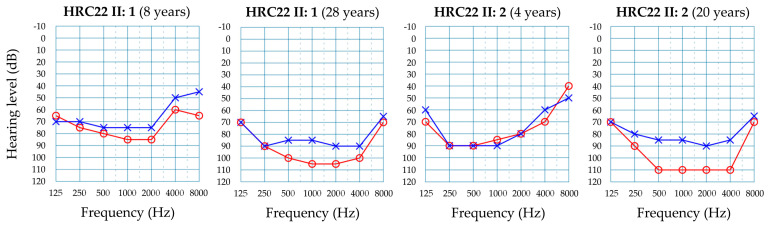
Audiograms from affected subjects from family HRC22 who carry *LARS2* variants causing sensorineural hearing loss. Only results for air conduction are shown. Red line and circles, right ear. Blue line and crosses, left ear. Audiograms at different ages, to illustrate the evolution of the hearing loss.

In Spanish family HRC22, the two affected brothers were compound heterozygous for c.308G>A (p.Arg103His) in exon 4 of *LARS2* and c.795A>G (p.Ile265Met) in exon 9, whereas their father carried c.308G>A and their mother carried c.795A>G (Figure 5). The family had no siblings with normal hearing. The c.308G>A missense variant was reported previously in the compound heterozygous state with c.1552G>A (p.Asp518Asn) in a male subject with reversible myopathy, lactic acidosis and developmental delay [18]. The c.795A>G missense variant is novel. It affects a conserved residue in the catalytic domain of the LARS2 polypeptide (Figure 5c) [15]. According to the ACMG criteria, the variant is classified as likely pathogenic (Table 2). In both affected brothers of family HRC22, the hearing loss was prelingual and severe, low and middle frequencies being more affected (Figure 6). Over the years, it progressed to become profound with a ‘cookie-bite’ audiogram. No other clinical signs were found in their medical histories or in their reexamination at the time of genetic diagnosis.

Table S2 summarizes the clinical features of all affected subjects in this report.

## 4. Discussion

Biallelic pathogenic variants in the *KARS1*, *HARS2* and *LARS2* genes, which encode the mtARS for lysine, histidine and leucine, respectively, result in a great variety of phenotypes ranging from NSHI to very complex syndromes, with a predominance of neurological signs [3,15]. Given that these enzymes play not only their catalytic role but also some other functions, which they perform through different protein domains, and given the predominance of missense mutations that affect those different domains in a combinatorial way, it is not surprising that establishing genotype–phenotype correlations is a formidable task. Here, we report five cases with different pathogenic variants in those genes, five of them novel, which have resulted only in NSHI, as no other clinical signs have been detected to date.

Specifically, the consequences of pathogenic variants in *KARS1* gene are more difficult to analyze, as the gene encodes both the cytosolic and mitochondrial ARS for lysine. So most pathogenic variants affect both types of isoforms, multiplying their possible effects. As other mtARS, the mitochondrial KARS1 polypeptide contains N-terminal mitochondrial targeting sequences (MTS, absent in the cytosolic isoform), an anticodon-binding domain and a catalytic domain. It also contains a helix of dimerization (residues 236–262), as KARS1 belongs to ARS class II, and it forms mainly dimers, although a tetrameric structure is also possible [15,19]. In addition, cytosolic KARS1 is a component of the multi-synthetase complex (MSC), that consists of ten tRNA synthases and three scaffold proteins. Over 50 pathogenic variants have been reported in *KARS1*, and most affected subjects have complex syndromes that can include hearing loss, peripheral neuropathy [20], optic neuropathy [21], cardiomyopathy [22] and leukoencephalopathy [5,23]. Only three unrelated cases have been reported to have NSHI (DFNB89 type) [4]. Two of them were homozygous for the same mutation, p.Tyr173His, in the anticodon-binding domain. The other one was homozygous for p.Asp377Asn, in the catalytic domain. In this work, we add two unrelated familial cases with NSHI: compound heterozygotes for p.Arg256Cys (in the helix of dimerization) and p.Thr358Met (in the catalytic domain) in family HRC19; and homozygotes for p.Ile294Thr (also in the catalytic domain) in family HRC20. Of note, p.Thr358Met (homozygous) and p.Ile294Thr (in compound heterozygous state with p.Thr587Met) have been reported in syndromic cases [14,16]. Therefore, in our two cases, the more benign phenotype may be attributed to p.Arg256Cys and p.Ile294Thr. Structural analyses of the p.Tyr173His variant indicated that it does not cause any relevant conformational change in the protein, but it may trigger subtle changes in the anticodon-binding domain [24]. As the other variants putatively responsible for the NSHI phenotype lie in different domains, further structural and functional analyses are needed to elucidate their pathogenic mechanisms, and establish hypothetical differences with those of mutations leading to syndromic conditions. It has been postulated that those phenotypes that are restricted to some organs or tissues may result from specific high requirements of the involved amino acid in those organs or tissues. As regards *KARS1*, the human proteome was analyzed to identify proteins with percentages of lysine higher than the proteome average (6%) and then it was considered whether those proteins had roles in the inner ear. The TMIE protein, with 15% lysine, whose gene is involved in DFNB6 NSHI, was found among them [25]. Further work is needed to investigate these hypothetical specificities. Nevertheless, it must be kept in mind that there is a great interfamilial and intrafamilial phenotypic variability in the syndromic cases, with different expressivity of the diverse clinical signs [14]. Also, subjects with DFNB89 NSHI should be followed up, as other clinical signs may manifest later.

In many subjects with pathogenic variants in the genes encoding mtARS, the hearing phenotype has not been extensively described. Here, we provide pure-tone audiograms from all the cases who were elucidated in this study. As regards *KARS1*, the three previously reported unrelated cases with NSHI had slightly downsloping audiograms, the hearing loss being moderate, severe and profound, respectively, and prelingual in all cases [4]. In family HRC19, the hearing loss was also prelingual, but in family HRC20 it was postlingual. The shape of the audiograms in HRC19 and HRC20 shows intrafamilial similarity, but interfamilial variability (‘cookie-bite’ versus flat or mildly downsloping). In both families the hearing loss is initially moderate, and a mild progression was documented in family HRC19 over the years, whereas the progression was extremely rapid in HRC20. Of note, in one syndromic case in the literature, the hearing loss was prelingual and profound, with an audiogram reminiscent of a ‘cookie-bite’ shape [5].

Biallelic pathogenic variants in *HARS2* and *LARS2* result in Perrault syndrome, which associates hearing impairment with ovarian dysfunction and, occasionally, a variety of neurological signs. In consequence, it can manifest just as another form of NSHI in males, as well as in females in their early stages of life. This is the situation we have observed in the three unrelated cases that we report in this study (2 *HARS2*, 1 *LARS2*). Taking into account the three novel mutations in our study, 20 different *HARS2* pathogenic variants have been reported to date [15,26], including in this work. Only three of them (including the novel p.Arg159* that is reported here) are truncating mutations, an expected fact as complete knockout of any ARS has been shown to be embryonically lethal in different model organisms [27]. Of note, all three truncating mutations were compound heterozygous with the same variant, the recurrent p.Arg480His. Interestingly, subjects with biallelic *HARS2* pathogenic variants, like the five reported in this study, do not show neurological signs [26] or they may be very subtle [17,28], in contrast to what is observed for other genes involved in Perrault syndrome [26]. As regards ovarian dysfunction, our three female patients are still young (in the 21–24 years-old range), and hitherto they do not show any related sign. In the subjects with *HARS2* pathogenic variants who have been reported in the literature, the onset of hearing impairment occurs early in the infancy (prelingual), like in our five patients. It seems it starts as a severe hearing loss that quickly progresses to become profound [17,28,29,30]. Audiograms from subjects HRC21 II:2 and II:3 illustrate this progression (II:2 more slowly), whereas HRC21 II:4 and both affected sisters of family 491NS had an earlier and quicker evolution. This rapid progression of the hearing loss was also observed in a targeted knockout of *Hars2* in mouse cochlear hair cells [31].

Over 30 pathogenic variants have been reported in *LARS2* [15]. The predominant phenotype seems to be Perrault syndrome without additional features (NSHI in males), like in the two brothers of family HRC22 [9,32,33,34,35]. However, there are also reports of patients with developmental delay [36], leukodystrophy [18,37], reversible mitochondrial myopathy [18] and Hydrops, Lactic Acidosis and Sideroblastic Anemia (HLASA) [18]. Again, it is not possible to establish genotype–phenotype correlations, as some mutations, like p.Arg103His, are shared by compound heterozygous patients with only NSHI (HRC22 II:1 and II:2) or with complex syndromes (reversible mitochondrial myopathy and developmental delay) [18], the difference hypothetically being in the accompanying variation in the other allele. In the two affected brothers of family HRC22, their hearing loss has a prelingual onset, and it starts being severe with an upsloping audiogram. Later it progresses to become profound, with low and middle frequencies being more severely affected. A similar pattern has been reported in several patients from the literature [32,33,35], suggesting it could be a distinctive feature of *LARS2*-associated hearing loss.

## 5. Conclusions

Establishing genotype–phenotype correlations for pathogenic variants affecting the genes encoding mitochondrial ARS is a complex task that still requires reports of many more cases with a thorough clinical characterization. Despite these difficulties, some patterns are beginning to emerge: (i) early onset and quick progression of the hearing loss (from moderate/severe to profound) seem to be common features; (ii) middle frequencies are more severely affected, the ‘cookie-bite’ audiogram being relatively frequent; (iii) *HARS2* pathogenic variants result in Perrault syndrome without neurological signs; (iv) the highest interfamilial and intrafamilial phenotypic heterogeneity is observed in *KARS1*, with a vast majority of diverse syndromic conditions. However, our results confirm the involvement of *KARS1* in DFNB89 NSHI, for which until now there was limited evidence, given the few described cases. Finally, our data indicate that although the contribution of *KARS1*, *HARS2, LARS2* and *NARS2* is quantitatively small (5 out of 193 non-DFNB1 cases (2.6%) taking the Spanish and Colombian cohorts and the four genes in aggregate), they must be included in targeted gene-sequencing panels, as hearing loss is the first clinical sign that the patients manifest and their early genetic diagnosis will improve their follow up and management.

## Figures and Tables

**Table 1:** Primers and PCR conditions for amplicons containing pathogenic variants.

Gene	Exon(s)	Primer Sequences (5′-3′)	Annealing T (°C)
*KARS1*	6–7	Upper: TCCTTCTTGGGCTCACATTAAC Lower: TGACACAGGATACGCTTTGG	60
	8	Upper: TCATAGTGTCCTTTTTTGTC Lower: TCTCTCTGTTCCTCTTATTTC	60
	9	Upper: CAAGAGCAAAACTCCATCTCA Lower: CTGCCTGTAATATGCTTCACC	60
*HARS2*	5–6	Upper: CCAGTCCTCCCTAATGTC Lower: AACAATGGCAGTACCTACTTT	55
	7	Upper: GTCAGGAAAGTAGGTACTGCC Lower: AAATAGCCCACTTCCAGC	55
	9–10	Upper: CTAGAGCACATTAGGGATAA Lower: CAATTCCAAAAATCAAGA	55
	11–12	Upper: GTTTCTGGAGGTGTAGTTGGA Lower: ACAGGCAGCAATGGTCAT	55
*LARS2*	4	Upper: TTGAATTTTGCTTTTTGACATT Lower: CAGCACAACACCCAACTC	55
	9	Upper: TGGATCTTCTTTAGTGTCCC Lower: CCCAAGTTGCTGGTTTAC	55

**Table 2:** Assessment of pathogenicity of the novel missense variants in this study.

Gene ^1^	Variant		SIFT Score	Polyphen-2 Score	CADD Score	REVEL Score	Minor Allele Frequency (MAF)	ACMG Criteria	Classification
	DNA	Protein							
*KARS1*	c.766C>T	p.Arg256Cys	0	1.000	35	0.744	4 × 10^−6^ (global) 6 × 10^−6^ (European non-Finnish)	PM2 (moderate), PM3 (strong), PP1 (moderate) PP3 (supporting)	Likely pathogenic
*HARS2*	c.728A>C	p.Asp243Ala	0.001	0.885	23.8	0.317	8 × 10^−6^ (global) 7 × 10^−5^ (Admixed American)	PM2 (moderate), PM3 (strong), PP1 (moderate)	Likely pathogenic
*HARS2*	c.1012G>A	p.Glu338Lys	0.001	0.999	32	0.871	6 × 10^−5^ (global) 0 (Admixed American)	PM2 (moderate), PM3 (strong), PP1 (moderate) PP3 (moderate)	Pathogenic
*LARS2*	c.795A>G	p.Ile265Met	0.001	0.987	20.4	0.556	8 × 10^−6^ (global) 3 × 10^−6^ (European non-Finnish)	PM2 (moderate), PM3 (strong), PP1 (moderate)	Likely pathogenic

^1^ Accession numbers: *KARS1*, NM_001130089.2; *HARS2*, NM_012208.4; *LARS2*, NM_015340.4.

## Data Availability

Data on the novel pathogenic variants that are reported in this study are available in ClinVar (accession numbers SCV005061478 to SCV005061482).

## Supplementary Materials

The following supporting information can be downloaded at: https://www.mdpi.com/article/10.3390/genes15070951/s1, Figure S1: Scheme depicting the structure of the mtARS whose genes were investigated in this work; Table S1: Genes that were investigated in this work, and their associated diseases; Table S2: Summary of the clinical features of the affected subjects.
