# Supplementary material for: Novel Cases of Non-Syndromic Hearing Impairment Caused by Pathogenic Variants in Genes Encoding Mitochondrial Aminoacyl-tRNA Synthetases
Source: Genes (Basel). 2024 Jul 19;15(7):951. doi: 10.3390/genes15070951 (PMC11276111; doi:10.3390/genes15070951)
Supplement: Supplementary file 1 [file genes-15-00951-s001.zip › genes-3097346-supplementary.pdf]

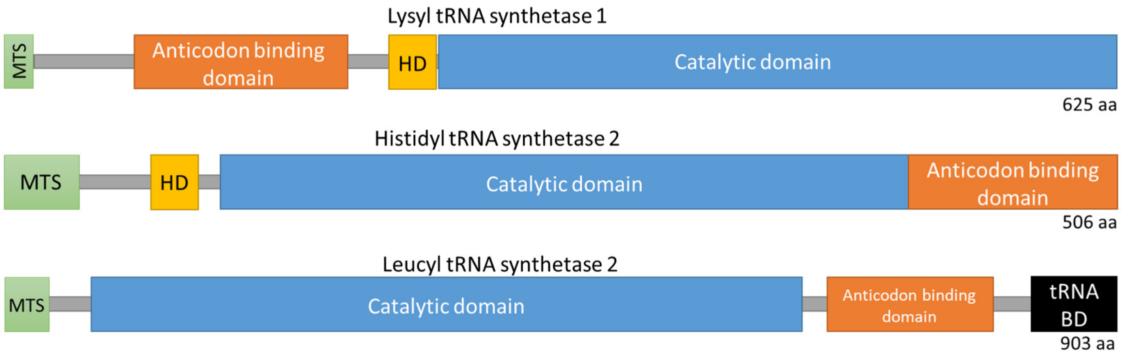

Figure S1. Scheme depicting the structure of the mtARS whose genes were investigated in this work, as indicated in the MiSynPat database [15]. MTS, Mitochondrial Targeting Signal; HD, Helix of Dimerization; tRNA BD, tRNA Binding Domain.

Table S1. Genes that were investigated in this work, and their associated diseases.

Gene	OMIM	Disease	OMIM
KARS1	601421	Deafness, autosomal recessive 89 (DFNB89)	613916
		Deafness, congenital, and adult-onset progressive leukoencephalopathy	619196
		Leukoencephalopathy, progressive, infantile-onset, with or without deafness	619147
HARS2	600783	Perrault syndrome 2	614926
LARS2	604544	Perrault syndrome 4	615300
		Hydrops, lactic acidosis, and sideroblastic anemia	617021

Table S2. Summary of the clinical features of the affected subjects.

Subject	Gene	Genotype	Hearing loss	Comorbidities
HRC19 II:1	*KARS1*	p.Arg256Cys/ p.Thr358Met	Prelingual, 'cookie-bite' audiogram, mildly progressive (moderate to severe)	None
HRC19 II:2	*KARS1*	p.Arg256Cys/ p.Thr358Met	Prelingual, 'cookie-bite' audiogram, mildly progressive (moderate to severe)	None
HRC20 II:3	*KARS1*	p.Ile294Thr/ p.Ile294Thr	Postlingual onset (40 y), moderate, sudden progression to profound	Fluctuating dysgeusia (metallic taste)
HRC20 II:4	*KARS1*	p.Ile294Thr/ p.Ile294Thr	Postlingual onset (15 y), moderate, sudden progression to profound	None
HRC21 II:2	*HARS2*	p.Arg159*/ p.Arg480His	Prelingual, progressive, from moderate-severe (slightly downsloping audiogram) to profound ('cookie-bite' audiogram)	None
HRC21 II:3	*HARS2*	p.Arg159*/ p.Arg480His	Prelingual, progressive, from moderate-severe (slightly downsloping audiogram) to profound ('cookie-bite' audiogram)	None
HRC21 II:4	*HARS2*	p.Arg159*/ p.Arg480His	Prelingual, progressive, profound ('cookie-bite' audiogram)	None
491NS II:1	*HARS2*	p.Asp243Ala/ p.Glu388Lys	Prelingual, profound, flat audiogram	None
491NS II:2	*HARS2*	p.Asp243Ala/ p.Glu388Lys	Prelingual, profound	None
HRC21 II:1	*LARS2*	p.Arg103His/ p.Ile265Met	Prelingual, progressive, from severe (low and middle frequencies) to profound ('cookie-bite' audiogram)	None
HRC21 II:2	*LARS2*	p.Arg103His/ p.Ile265Met	Prelingual, progressive, from severe (low and middle frequencies) to profound ('cookie-bite' audiogram)	None
